# Surgical treatment of arteriovenous malformations of the buttock

**DOI:** 10.1016/j.jvscit.2022.05.008

**Published:** 2022-07-01

**Authors:** Claude Laurian, Nikos Paraskevas, Michele Bigorre, Claudine Masonni, Pierre Cerceau, Francesca Toni, Annouck Bisdorff

**Affiliations:** a Department of Vascular Surgery, Saint Joseph Hospital, Paris, France; b Department of Vascular Surgery, Bichat Hospital, Paris, France; c Department of Plastic Surgery, Arnaud de Villeneuve Hospital, Montpellier, France; d Ultrasonography Center, Paris, France; e Alma Clinic, Paris, France; f Department of Neuroradiology, Lariboisère Hospital, Paris, France

**Keywords:** Arteriovenous malformation, Embolization, Gluteal area, Surgical resection

## Abstract

For symptomatic buttock arteriovenous malformations (AVMs), embolization techniques and surgical resection have been suggested as treatment options. Our aim was to evaluate the feasibility and long-term results after a single surgical resection. Twelve patients had undergone surgical resection without preoperative embolization. Of the 12 patients, 11 had had incomplete procedures, 9 of whom had undergone arterial embolization 1 to 3 years previously. All the patients were symptomatic. Computed tomography scans showed AVMs located in the cellular spaces with preservation of the gluteal muscle. The median follow-up time was 80 months. On the last follow-up computed tomography scan, 67% had had no residual AVM. The use of preoperative embolization, especially with nonresorbable embolic material (Onyx; Medtronic, Dublin, Ireland), makes AVM resection and imaging follow-up more difficult because of artifacts and should be avoided.

Arteriovenous malformations (AVMs) of the buttock are in an unusual location and can result in severe complications. Our understanding of AVMs has evolved from an angiographic concept with the identification of a nidus between feeding arteries and draining veins to a tissular concept with the identification of a specific tissue in which the nidus develops.

Reported studies of surgical resection have been limited with various surgical difficulty and incomplete results.^1^, ^2^, ^3^ Thus, numerous techniques of interventional radiology have been proposed, including arterial, transvenous, and percutaneous embolization.^4^, ^5^, ^6^, ^7^, ^8^ However, the use of radiologic procedures can never cure the underlying AVM. The use of nonresorbable embolic agents (eg, Onyx; Medtronic, Dublin, Ireland) has resulted in a new concern because of their disadvantages such as the creation of artifacts during ultrasound (US) and computed tomography (CT) examinations.^4^ The peculiarity of buttock AVMs is the anatomic extent of the nidus in the cellular spaces with respect to the musculoskeletal structure. The aim of the present study was to demonstrate the clinical and anatomic results after a single surgery without the use of preoperative embolization.

## Methods

We retrospectively reviewed 12 consecutive patients who had undergone surgical treatment for an AVM of the buttock between 1998 and 2018. All the patients or their guardians provided oral and written informed consent for the treatment and provided consent for the report of their case details and imaging studies.

Patients were included in the study if they had had symptoms despite medical treatment or after the lack of improvement after previous embolization. All the patients had undergone digital radiography, US, and CT with arterial reconstruction. Preoperative arterial embolization was chosen only for patients with acute bleeding.

## Results

**Fig 1:**
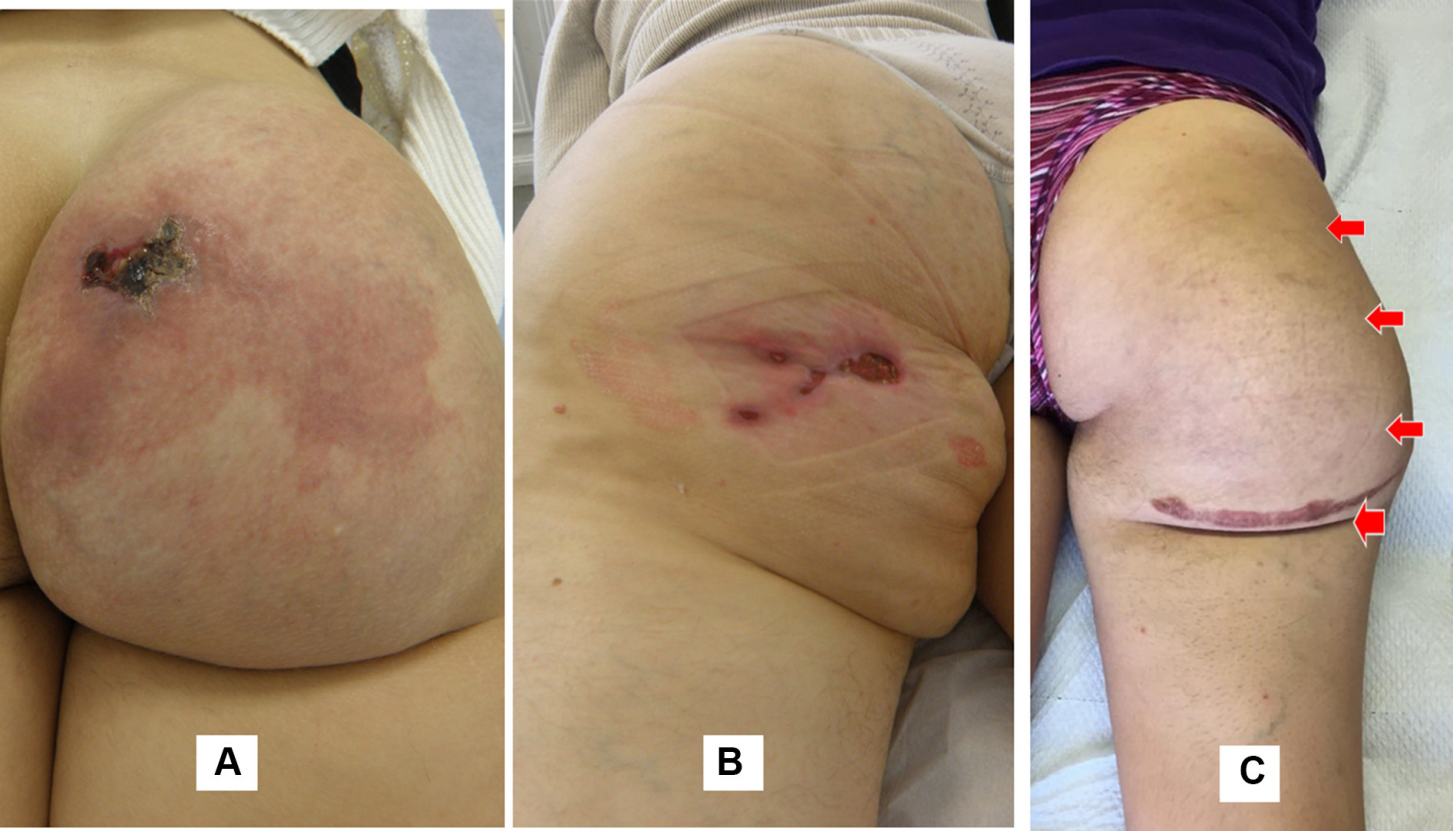
Clinical aspects of arteriovenous malformation (AVM) of the buttock. **A,** False port-wine stain with skin ulceration. **B,** Ischemic skin lesions after embolization. **C,** Swelling of the buttock with pulsatile mass, after previous surgical exploration.

Twelve patients underwent surgery (6 females and 6 males; mean age, 27 years; range, 15-42 years). Eleven had previously undergone an incomplete procedure 1 to 3 years before inclusion in the study. All the patients were symptomatic and had presented with pain in 12 patients, a pulsatile mass in 12), a port wine stain (PWS) in 6, skin ulceration in 7, and recurrent bleeding in 4 (Fig 1). One of them had had acute bleeding and underwent emergency embolization to treat the complication.

**Fig 2:**
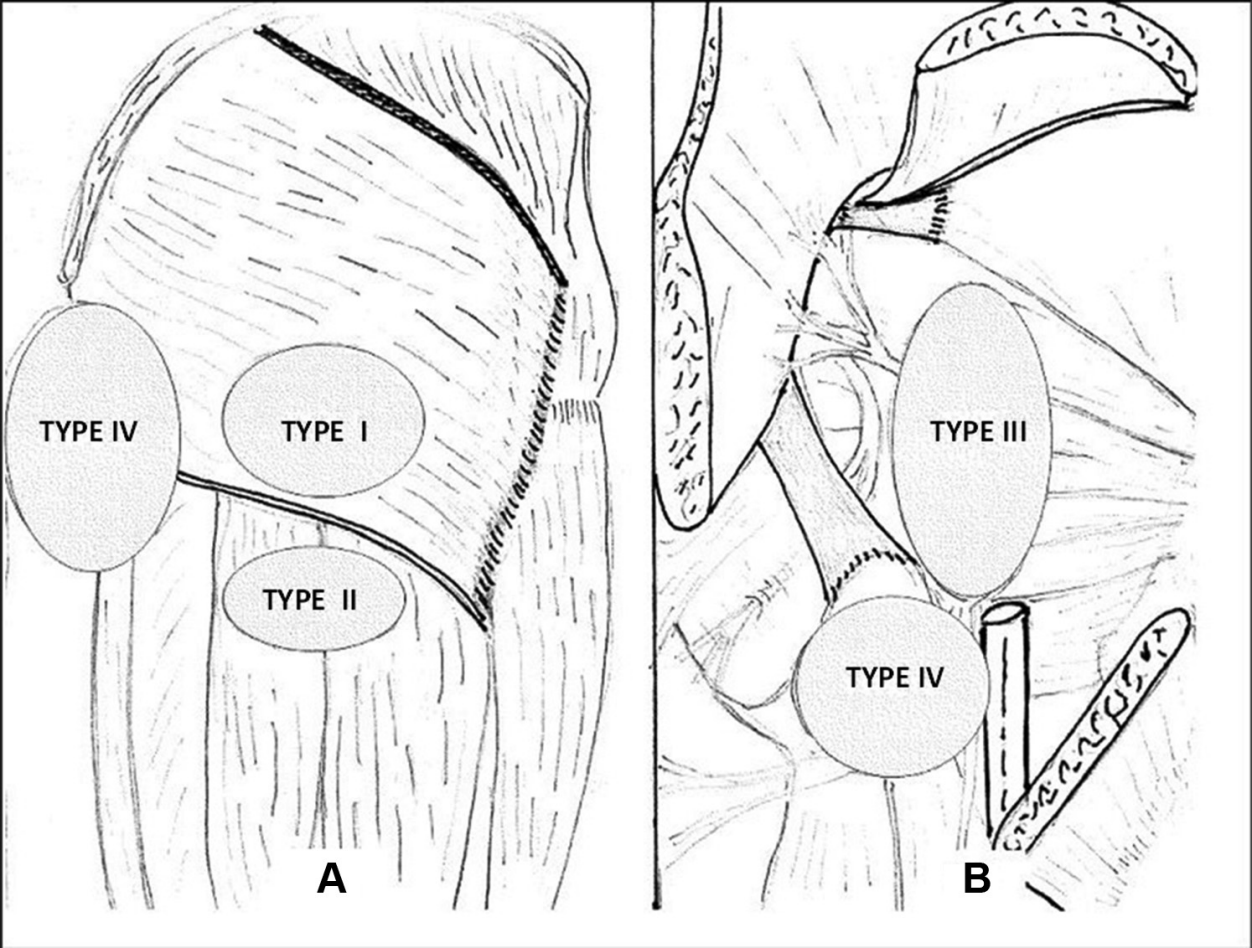
Location of arteriovenous malformation (AVM) in the cellular spaces of the buttock. **A,** Type I: superficial AVM, top gluteal fold. Type II: AVM bottom gluteal fold. **B,** Type III: AVM in the intermuscular space between gluteal muscles. Type IV: AVM overlying and underlying the gluteus maximus muscle.

The mean blood flow of the AVM on US was 1640 mL/min (range, 900-3500 mL/min). The CT scan was used to evaluate the location of the nidus in the cellular space, allowing four anatomic areas to be distinguished (Fig 2). The four anatomic areas were as follows: type I, a well-localized AVM, including cutaneous, subcutaneous tissue, and fascia overlying the gluteus muscle of the buttock; type II, AVM extent in the subcutaneous tissue in the upper thigh; type III, AVM in cellular space between the gluteus muscles; and type IV, AVM extension into the cellular space overlying and underlying the gluteus maximus muscle (ischiorectal fossa). This classification represents the dominant clinical aspect at inclusion but two types can be associated initially or before reoperation for a residual AVM (3 patients). For open surgical treatment, the particularities of this anatomic area mean that the surgical approach will depend on the extent of the AVM and that staged surgical resection could be an option.

**Fig 3:**
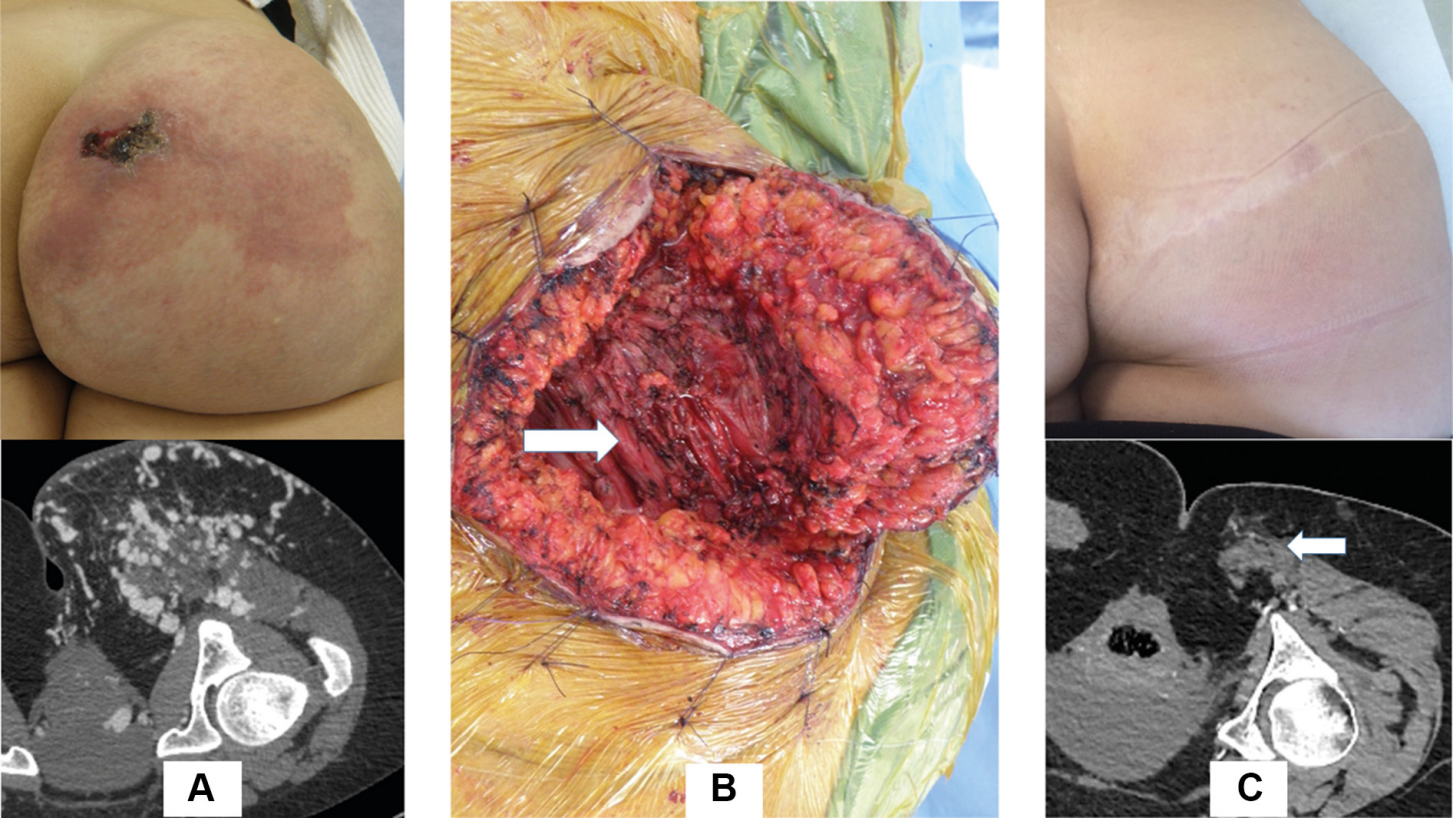
**A,** Preoperative photograph and computed tomography (CT) scan of an arteriovenous malformation (AVM). **B,** Intraoperative photograph of an AVM (*arrow*). **C,** Postoperative photograph and CT scan showing the repaired AVM (*arrow*).

The different approaches were as follows. For superficial AVMs, large resection of the PWS or excess tissue was performed using diamond cutaneous and subcutaneous resection (Fig 3). Intermuscular AVMs can require an incision in the gluteal fold that continues to the lateral buttock, permitting the gluteus maximus muscle’s tendon to be cut and the AVM exposed in the anterior part of the muscle. Complex AVMs on the inferior medial quadrant will require an incision in the gluteal fold that continues with a vertical incision to the medial part of the buttock with exposure of the gluteal inferior pedicle.

The dominant cellular spaces found to be affected at the first evaluation included superficial AVMs with a false PWS, type I (7%-67%); cellular space in the upper thigh, type II (2 patients); intermuscular cellular space, type III (2 patients); and cellular space around the gluteus muscle (1 patient). Surgery was incomplete in five patients owing to extent of the AVM (type IV) around the inferior border of the gluteal muscle.

Complete resection within one session was possible for seven patients (58.3%) and was incomplete for five patients (41.6%). In all 12 patients, the gluteus muscle was preserved. Five patients had required an intraoperative or postoperative blood transfusion. The mean blood loss was 550 mL (range, 200-1500 mL). The median operative time was 6 hours (range, 5-11 hours).

In the early postoperative period, four patients had presented with delayed healing. One patient had required reoperation for a hematoma. After the 6-month follow-up, six patients had had no clinical residual AVM, two had presented with a new pulsatile mass at 2 months, and four had had a residual AVM identified on a follow-up CT scan.

At the last follow-up (median, 80 months; range, 20-216), 8 of the 12 patients had reported no residual pain. One patient was lost to follow-up at 12 months. Of the 12 patients, 6 had required additional surgery. Three had required surgery for a residual AVM (two had required three surgical sessions and one had required two surgical sessions), three had required plastic surgery, and two had required surgery to treat extrusion of an embolic agent.

**Fig 4:**
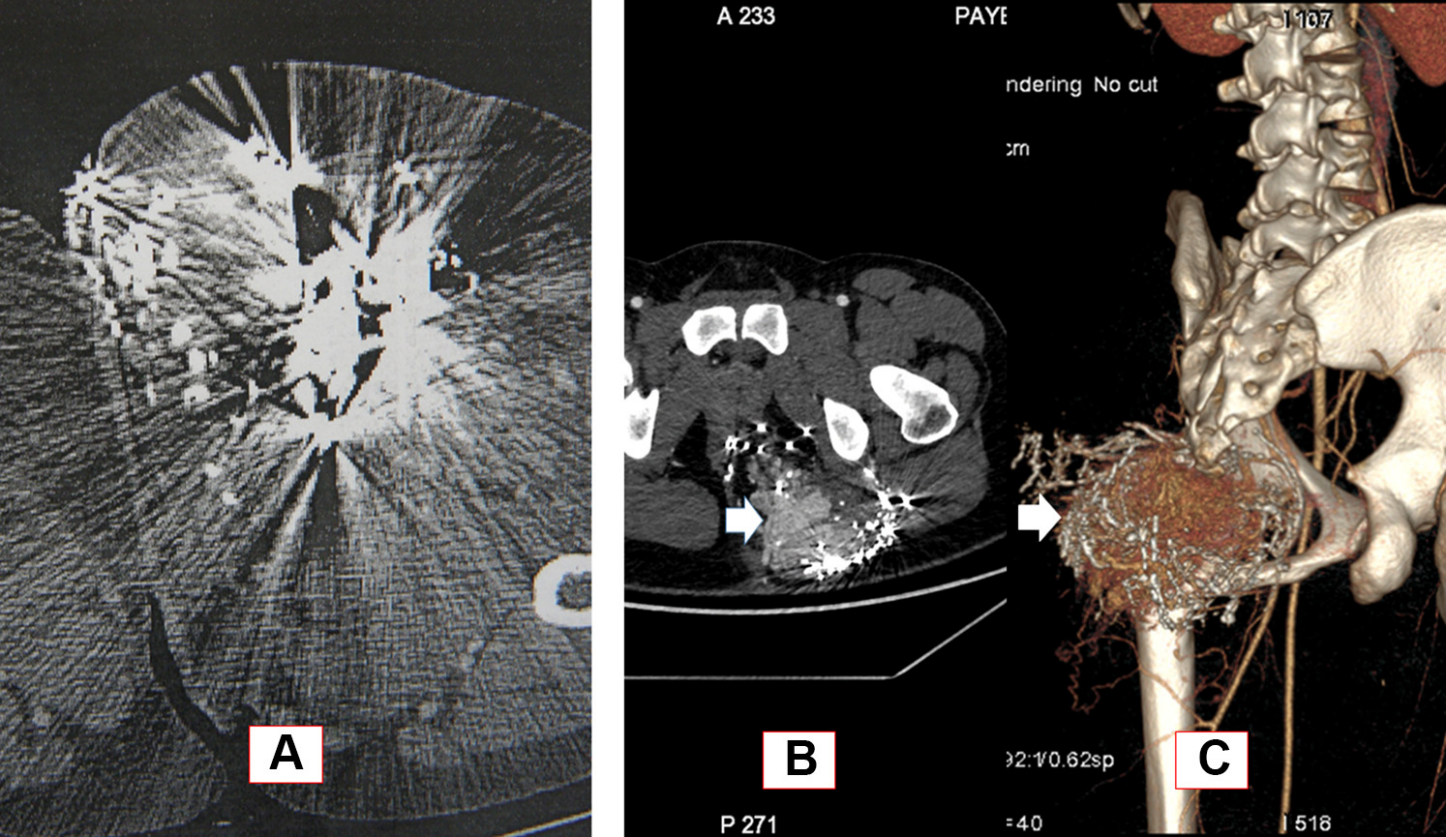
Disadvantadges of embolization with Onyx agent. **A,** MRI after embolization with Onyx and diffuse artefacts. **B** and **C,** Residual arteriovenous malformation (AVM) after Onyx embolization, with a ring of Onyx around the residual nidus in the ischio-rectal fossa.

Follow-up CT scans had been performed at 6 to 12 months postoperatively for all 12 patients (100%). Of the 12 patients, 8 (67%) showed no residual AVM, 2 had had symptomatic residual AVMs, and 2 had had asymptomatic AVMs. Of the 12 follow-up CT scans, 2 had had numerous artifacts owing to the use of a nonresorbable embolic agent (Fig 4).

## Discussion

In the present report, we have described our findings for 12 patients who had had an AVM located in the buttock. A few previous studies have reported on surgical treatment of AVMs of the buttock.^1^^,^^2^^,^^4^ One study had reported that the location of the AVM was in the connective tissue.^1^

The mainstays of therapy have included medical management, embolization, and surgical resection. The use of embolization resulted from advances in interventional radiology, which have had an effect on the treatment of complications after percutaneous embolization and stabilizing AVM progression.^4^^,^^5^ However, the incomplete effectiveness of such treatment on the underlying AVM has persisted.^6^, ^7^, ^8^ In our cohort, 9 of the 12 patients (75%) had previously undergone embolization. The patients had not experienced clinical benefits in terms of relief of pain or swelling. Two patients had developed skin necrosis (one with extrusion of the embolic agent), and two had developed recurrent skin ulceration with bleeding. We observed the deleterious consequences of embolization with a nonresorbable agent (Onyx; Medtronic) in two patients with nontarget embolization (ischemic skin lesions) and artifacts on the US and CT follow-up examinations.

For surgical resection, mapping with US guidance can be used to locate the AVM target. Next, a surgical approach can be chosen according to the cellular space involved. Of our 12 patients, 7 had required only a single surgical resection. A staged resection was performed for AVMs that had involved multiple anatomic areas. Previous studies have reported the inadequate outcomes of surgery with the risk of intraoperative bleeding. In our study, the mean blood loss was 550 mL.

A residual AVM can be present after a “curative” procedure. A residual AVM can result from inaccurate preoperative mapping or inadequate exposure of the lesions owing to the surgical approach. The final CT scan showed that 4 of our 12 patients (33%) had had a residual AVM, 2 of whom were scheduled for an additional procedure.

Neither the clinical classification nor the angiographic classification consider the involvement of the tissue.^9^^,^^10^ Our indications for surgery included an AVM with recurrent bleeding, persistent skin ulcerations, or pain. For AVMs located in the buttocks, the arterial feeders and draining veins will be numerous, which explains the palliative approach of the endovascular procedure. In addition, AVMs with acute bleeding will require an arteriographic evaluation with two treatment options: arterial or percutaneous embolization. Finally, a residual AVM should be treated by staged resection after precise recognition of the residual nidus via CT.

## Conclusions

Treatment of AVMs of the buttock can be very challenging. A CT examination offers precise identification of the tissue involved in the cellular space representing the specific target. Surgery is an invasive approach and should be used selectively. The long-term outcomes from our retrospective, single-center analysis suggest that surgical resection of AVMs is a valuable treatment option.

## References

[1] Wolley M.M., Stanley P., Wesley J.R. (1977). Peripherally located congenital arteriovenous fistulae in infancy and childhood. J Pediatr Surg.

[2] Shindo S., Tada Y., Shirakawa M., Takayama Y., Myata T., Sato O. (1990). Congenital arteriovenous fistula in the gluteal region: a report of five cases. Jap J Surg.

[3] Liu A.S., Mulliken J.B., Zurakowowski D., Fishman S.J., Green A.K. (2010). Extracranial arteriovenous malformations: natural progression and recurrence after treatment. Plast Reconstr Surg.

[4] Nassiri N., Cirillo-Penn N.C., Crystal D.T. (2017). Direct stick embolization of extremity arteriovenous malformations with ethylene vinyl alcohol copolymer. J Vasc Surg.

[5] Jin Y., Yang X., Hua C., Lin X., Chen H., Lin X. (2017). Ethanol embolotherapy for the management for refractory chronic skin ulcers caused by arteriovenous malformations. J Vasc Interv Radiol.

[6] Park K.B., Do Y.S., Kim D.I., Kim Y.W., Park H.S., Shin S.W. (2019). Endovascular treatment results and risk factors for complications of body and extremity arteriovenous malformations. J Vasc Surg.

[7] Chellah M.P., Do H.M., Zinn Z., Patel V., Jeng M., Khosla R.K. (2018). Management of complex arteriovenous malformations using a novel combination algorithm. JAMA Dermatol.

[8] Vaisnyte B., Vajauskas D., Palionis D., Nevidomskyte D., Misonis N., Bilkis V. (2013). Complicated congenital gluteal arteriovenous malformation with hemorrhage in pregnancy. Ann Vasc Surg.

[9] Cho S.K., Do Y.S., Shin S.W., Kim D.I., Kim Y.W., Park K.B. (2006). Arteriovenous malformations of the body and extremities: analysis of therapeutic outcomes and approaches according to a modified angiographic classification. J Endovasc Ther.

[10] Yakes W., Baumgartner I. (2014). Interventional treatment of arteriovenous malformations. Gefasschirurgie.

